# The Dose–Response Effect of Fluoride Exposure on the Gut Microbiome and Its Functional Pathways in Rats

**DOI:** 10.3390/metabo13111159

**Published:** 2023-11-17

**Authors:** Zhe Mo, Jian Wang, Xinyue Meng, Ailin Li, Zhe Li, Wenjun Que, Tuo Wang, Korto Fatti Tarnue, Xu Ma, Ying Liu, Shirui Yan, Lei Wu, Rui Zhang, Junrui Pei, Xiaofeng Wang

**Affiliations:** 1 Key Laboratory of Etiology and Epidemiology, Chinese Center for Disease Control and Prevention, Center for Endemic Disease Control, Education Bureau of Heilongjiang Province & National Health Commission (23618504), Institute for Fluorosis Disease Control, Harbin Medical University, Harbin 150081, China; zhmo@cdc.zj.cn (Z.M.); 102690@hrbmu.edu.cn (J.W.); 2019020210@hrbmu.edu.cn (X.M.); liailin@hrbmu.edu.cn (A.L.); 2021020138@hrbmu.edu.cn (Z.L.); 2021020163@hrbmu.edu.cn (W.Q.); 2021020139@hrbmu.edu.cn (T.W.); 20210200005@hrbmu.edu.cn (K.F.T.); 2022020080@hrbmu.edu.cn (X.M.); 2022020107@hrbmu.edu.cn (Y.L.); 2023020208@hrbmu.edu.cn (S.Y.); 2023020210@hrbmu.edu.cn (L.W.); 2023020215@hrbmu.edu.cn (R.Z.); 2 Department of Environmental Health, Zhejiang Provincial Center for Disease Control and Prevention, Hangzhou 310051, China

**Keywords:** fluoride exposure, gut microbiome, functional pathways, toxicity

## Abstract

Metabolic activities within the gut microbiome are intimately linked to human health and disease, especially within the context of environmental exposure and its potential ramifications. Perturbations within this microbiome, termed “gut microbiome perturbations”, have emerged as plausible intermediaries in the onset or exacerbation of diseases following environmental chemical exposures, with fluoride being a compound of particular concern. Despite the well-documented adverse impacts of excessive fluoride on various human physiological systems—ranging from skeletal to neurological—the nuanced dynamics between fluoride exposure, the gut microbiome, and the resulting dose–response relationship remains a scientific enigma. Leveraging the precision of 16S rRNA high-throughput sequencing, this study meticulously examines the ramifications of diverse fluoride concentrations on the gut microbiome’s composition and functional capabilities within Wistar rats. Our findings indicate a profound shift in the intestinal microbial composition following fluoride exposure, marked by a dose-dependent modulation in the abundance of key genera, including *Pelagibacterium*, *Bilophila*, *Turicibacter*, and *Roseburia*. Moreover, discernible alterations were observed in critical functional and metabolic pathways of the microbiome, such as D-lyxose ketol-isomerase and DNA polymerase III subunit gamma/tau, underscoring the broad-reaching implications of fluoride exposure. Intriguingly, correlation analyses elucidated strong associations between specific bacterial co-abundance groups (CAGs) and these shifted metabolic pathways. In essence, fluoride exposure not only perturbs the compositional equilibrium of the gut microbiota but also instigates profound shifts in its metabolic landscape. These intricate alterations may provide a mechanistic foundation for understanding fluoride’s potential toxicological effects mediated via gut microbiome modulation.

## 1. Introduction

Fluorine is an essential element for the normal growth and development of organisms. However, excessive exposure can lead to fluorosis [1]. Due to the uneven distribution of chemical elements on the Earth’s crust, some regions contain excessive fluoride, leading to endemic fluorosis among the local population primarily manifested as dental and skeletal fluorosis [2]. Additionally, it negatively affects human reproductive development, the liver and kidney, and the endocrine, nervous, and genetic systems [2,3,4]. Drinking water is the primary source of fluoride exposure. Over 20 countries worldwide have excessive fluoride concentrations in their groundwater [5]. In China, more than 70 million people are still at risk of high fluoride exposure [6].

Several mechanisms have been proposed for fluoride-induced diseases, which include stress pathways, signaling routes, cell cycle dysregulation, cell apoptosis, and epigenetic changes [7,8,9]. Increasing evidence suggests that disturbances in the gut microbiota and their subsequent effects on metabolism and physiological functions play a significant role in disease development [10]. Given fluoride’s toxicity, it is crucial to elucidate the impact of high fluoride exposure on the gut microbiota and its metabolism and functions. Excessive fluoride intake through drinking water induces an immune response, damages the structure of the caecum and rectum, inhibits the proliferation of intestinal epithelial cells, restricts glycoprotein secretion, decreases the distribution of goblet cells and hypertrophic cells, and alters the diversity and composition of the mouse gut microbiome [11,12,13]. An imbalance in the gut microbiota might compromise the integrity of the intestinal barrier and directly lead to diseases [14].

Studies on the effects of fluoride exposure on the gut microbiota have yielded inconsistent results. Research indicates that exposure to 100 mg/L fluoride increases the diversity and richness of the gut microbiome in Kunming and ICR mice [11,12]. However, other studies have found no significant changes in the composition and function of the gut microbiota in BALB/c mice exposed to a 4 ppm fluoride dose [7]. Population studies have shown that children with dental fluorosis in high-fluoride-exposure areas have slightly lower bacterial diversity and richness compared to normal groups [15]. Most current studies are mouse-based, although some research has confirmed that rats have a gut microbiota more similar to humans [16]. Moreover, the majority of studies focus on the effects of fluoride exposure on microbiota diversity and richness, with fewer investigating the dose–response relationship between fluoride exposure and the gut microbiota.

Building on preliminary research on the effects of drinking water fluoride on rat gut microbiota [17], this study aims to analyze the dose–response relationship of fluoride exposure in drinking water to the rat gut microbiota. Initially, 16S rRNA amplicon sequencing will be employed to observe changes in the gut microbiota and explore dose–response relationships. Subsequently, predictions on the metagenome’s functions and metabolic pathways [18] will be made to observe changes in these areas. Lastly, a comprehensive analysis of the gut microbiota’s composition and function will be conducted to determine the correlation between the microbial community and its functionality.

## 2. Materials and Methods

### 2.1. Animal Experiment

Specific-pathogen-free (SPF) male Wistar rats (4 weeks of age, body weight 127.96 ± 14.01 g) were purchased from Vital River Laboratories (Beijing, China). Rats were fed with a pelleted rodent diet (Beijing Keao Xieli Feed Co., Ltd., Beijing, China) and filtered water ad libitum and were maintained in the SPF animal facility of Harbin Medical University. A total of twenty-five rats were housed under maintained conditions of a 12:12 h light/dark cycle, constant environmental temperature of 23 ± 3 °C, and 40–70% relative humidity. All experiments were approved by the Ethics Committee of the Endemic Disease Control Center of Harbin Medical University. After 1 week of acclimation, rats were randomized into five groups (5 rats/group): control rats received distilled water alone, and distilled water with 25 mg/L, 50 mg/L, 100 mg/L, and 150 mg/L of NaF was administered to rats in 4 groups for 12 weeks, respectively. The dosages were chosen based on previous studies concerning environmental fluoride levels, the toxicities of fluorides, and the exposure level and duration in rodents (see Section 4). The feces of each rat were regularly obtained in a sterile tube after animal model realization and stored at −80 °C.

### 2.2. DNA Extraction and 16S rRNA Gene Sequencing

Total bacterial DNA was extracted from fecal samples using the environmental sample DNA extraction kit based on the manufacturer’s instructions. Its concentration was monitored on the Qubit PicoGreen fluorescence quantification system (Thermo Fisher Scientific Inc., Shanghai, China) and purity was detected using 1% agarose gels. The variable region 3 and 4 (V3/V4) of the 16S RNA gene was PCR-amplified and then detected using 2% agarose gels. The amplifications were purified, quantified, and pooled, and then sequenced on an Illumina MiSeq sequencer (Genergy Biotechnology, Shanghai, China).

### 2.3. Sequencing Data Analysis

The raw mate-paired fastq files were merged, quality-filtered, and demultiplexed to obtain the optimized sequences. USEARCH software verson 11 with UPARSE-OTU algorithm (http://www.drive5.com/usearch/manual/uparseotu_algo.html (accessed on 5 October 2023)) was applied to cluster the operational taxonomical units (OTUs) with a threshold of 97% sequence similarity, and the Ribosomal Database Project classifier was used for taxonomic identification.

### 2.4. Microbiome Co-Abundance Groups (CAGs)

The abundant genus or species were applied to construct co-abundance groups (CAGs). The function “cor” was used to calculate their Kendall correlations, which were visualized with hierarchical Ward clustering with a Spearman correlation distance metric to define CAGs with function “hcut” in the “factoextra” package. The best number of clusters was determined according to the number of significance using a pairwise Adonis test among each group of the Kendall correlation matrix using the “pairwise.adonis” function in the “pairwiseAdonis” package. The expression levels of CAGs were calculated based on the sum of relative abundance of the same CAGs.

### 2.5. Functional Prediction via Tax4fun2 Package

Alterations in the intestinal microbiome do not necessarily signify functional changes [19]. Hence, studying the functional changes resulting from fluoride exposure, rather than merely the taxonomic composition of the intestinal microbiota, becomes imperative for understanding its health impacts. We employed the “Tax4fun2” package in R 4.2.3 software to predict alterations in the functions of the intestinal microbiota [18]. Tax4Fun2, an upgraded version of the Tax4Fun package, enables rapid functional spectrum predictions of prokaryotes based on 16S rRNA gene sequences. By integrating user-defined genome information, it significantly enhances the accuracy and robustness of predictive functional profiles [18]. Firstly, user-supplied 16S rRNA gene sequences are searched against the 16S rRNA reference sequences via BLAST using the runRefBlast function [18]. 16S rRNA gene sequences are aligned with reference sequences provided by Tax4Fun2 to identify closely related sequences. Secondly, functional predictions are subsequently calculated using the makeFunctionalPrediction function. During this step, the OTUs table supplied by the user is summarized based on the results of the next-neighbor search. Based on these search results, the abundance of operational taxonomic units (OTUs) for each sample is summarized, creating an association matrix (AM) that includes the functional profiles of identified reference sequences from the 16S rRNA search. Predicted profiles are later summarized based on KEGG pathways. Only OTUs passing a defined similarity threshold (default = 97%) are considered in the functional prediction [18]. In brief, the amplicon sequence variants were applied to predict the abundance of gene families (function) and MetaCyc pathways.

### 2.6. Statistical Analysis

The differences of abundances in the gut microbiome of phylum among groups were detected using an ANOVA test, and the pairwise differences between groups were identified via Tukey correction. Linear regression was applied to explore the relationship between fluoride exposures and gut microbiome/CAGs/functions, and then expressed as Pearson correlations. Spearman correlations between abundance in CAGs and MetaCyc pathways were performed to identify the association. All statistical analysis was conducted in R 4.2.3 software (R Foundation for Statistical Computing, Vienna, Austria). A two-tailed *p* value < 0.05 was defined as statistically significant.

## 3. Results

### 3.1. Distribution of the Gut Microbiome with Fluoride Exposure

**Figure 1:**
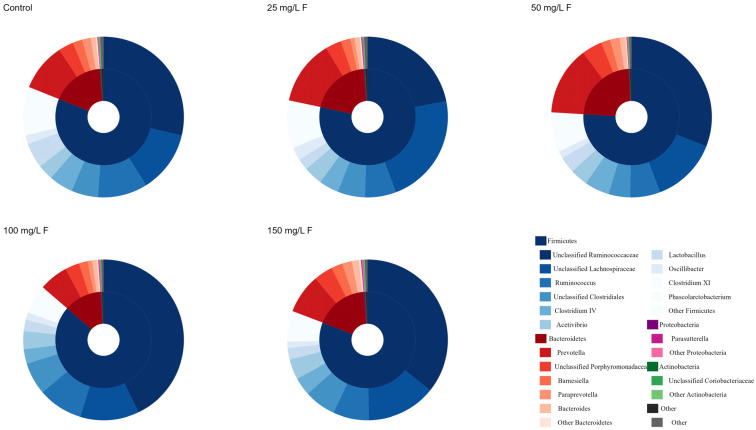
Mean relative abundance of the most abundant phylum and genus in the rats’ gut microbiome composition.

The gut microbiomes among the control and different NaF groups were dominated by taxa belonging to Firmicutes, Bacteroidetes, Proteobacteria, and Actinobacteria in descending order (Figure 1 and Table 1). A significant difference was only observed in the abundances of Proteobacteria among groups (*p* value = 0.043), and the result of the pairwise test showed that the 150 mg/L F group may have higher abundances than the 50 mg/L F group (adjusted *p* value = 0.046).

### 3.2. Influence of Fluoride Exposure on Gut Microbiome in Genus

**Figure 2:**
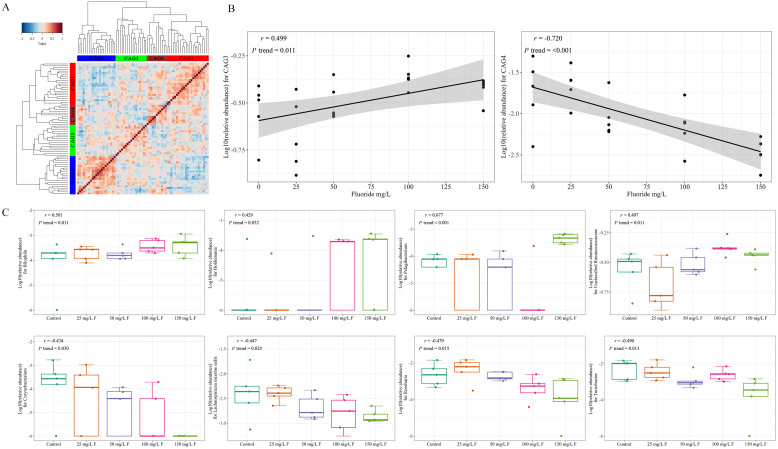
(**A**) Kendall correlations between genera clustered using hierarchical Ward method with a Spearman correlation distance metric. The primary genera attributed to the most CAGs are (proportion > 10%) CAG1 (*Unclassified Lachnospiraceae*, *Ruminococcus*, and *Unclassified Clostridiales*), CAG2 (*Prevotella* and *Unclassified Porphyromonadaceae*), CAG3 (*Unclassified Ruminococcaceae*), and CAG4 (*Turicibacter*, *Lachnospiracea incertae sedis*, *Roseburia*, and *Clostridium sensu stricto*). (**B**) The linear association between fluoride exposure and CAGs. (**C**) Boxplots show the effect of fluoride exposure on the gut microbiome in each genus with the Pearson correlation.

To clearly identify differences in gut microbiome structure among fluoride exposure groups, co-abundance correlations were clustered into four groups based on an Adonis test (Figure 2A). Significant associations were not observed between CAG1/CAG2 and fluoride exposure (*p* value > 0.05). CAG3 has a positive association with fluoride exposure, which means the relative abundance of CAG3 rises with increasing fluoride exposure doses, while CAG4 was negatively associated with fluoride exposure, which means the relative abundance of CAG4 decreases with increasing fluoride exposure doses (Figure 2B). Genera in *Bilophila*, *Holdemania*, *Pelagibacterium*, and *Ruminococcaceae* from CAG3 (Figure 2C and Table S1) were also found to be positively correlated with fluoride exposure, while *Corynebacterium*, *Lachnospiracea incertae sedis*, *Roseburia*, and *Turicibacter* from CAG4 (Figure 2C and Table S1) were negatively correlated with fluoride exposure.

### 3.3. Effect of Fluoride Exposure on Gut Microbiome in Species

**Figure 3:**
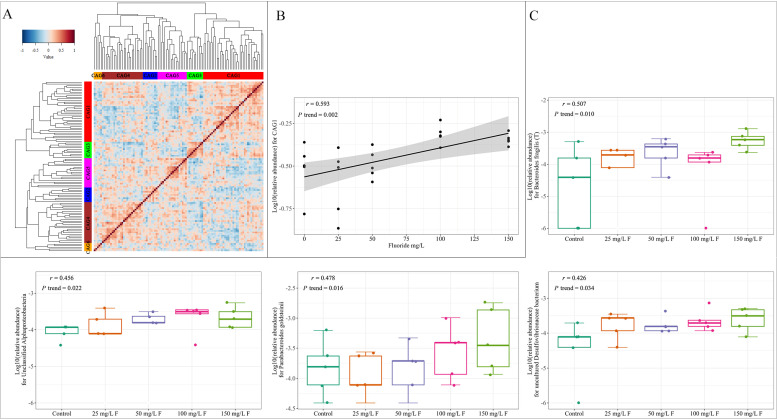
(**A**) Kendall correlations between species clustered using hierarchical Ward method with a Spearman correlation distance metric. The primary species attributed to the most CAGs are (proportion >10%) CAG1 (*Unclassified Ruminococcaceae*), CAG2 (*Uncultured rumen bacterium*), CAG3 (*Unclassified Prevotella*, *Uncultured bacterium* and *Unclassified Porphyromonadaceae*), CAG4 (*Uncultured Firmicutes bacterium*, *Rumen bacterium NK4A214* and *Unclassified Clostridium IV*), CAG5 (*Unclassified Lachnospiraceae*), and CAG6 (*Ruminococcus* sp., *Uncultured bacterium adhufec* and *Unclassified Blautia*). (**B**) The linear association between fluoride exposure and CAGs. (**C**) Boxplots show the effect of fluoride exposure on the gut microbiome in each species with Pearson correlation (due to space limitations, only some figures are shown).

Co-abundance correlations were clustered into six groups based on the Adonis test (Figure 3A). With the exception of CAG1, no significant association with fluoride exposure was observed in the other CAGs. CAG1 has a positive association with fluoride exposure, which means the relative abundance of CAG3 rises with increasing fluoride exposure doses (Figure 3B). Species in *Unclassified Pelagibacterium*, *Candidatus Soleaferrea massiliensis*, *Parabacteroides distasonis*, *Uncultured bacterium adhufec108*, *Unclassified Bdellovibrionales*, *Unclassified Ruminococcaceae*, *Bacteroidales bacterium ph8*, *Bacteroides fragilis (T)*, *Uncultured Kopriimonas* sp., *Unclassified Odoribacter*, *Parabacteroides goldsteinii*, *Unclassified Alphaproteobacteria*, *Bacteroides uniformis*, *Uncultured Erysipelotrichales bacterium*, and *Uncultured Desulfovibrionaceae bacterium* from CAG1 (Figure 3C and Table S2) were also found to be positively correlated with fluoride exposure.

### 3.4. Functional Changes in the Gut Microbiome with Fluoride Exposure

**Figure 4:**
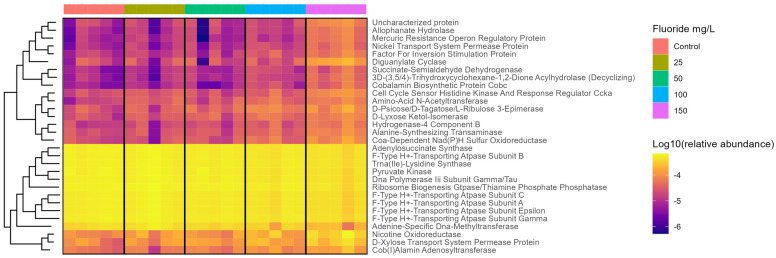
The relative abundance of the most correlated function (the absolute of Spearman correlation coefficient > 0.75 and *p*-value < 0.05) with fluoride exposure visualized via Heatmap plot.

**Figure 5:**
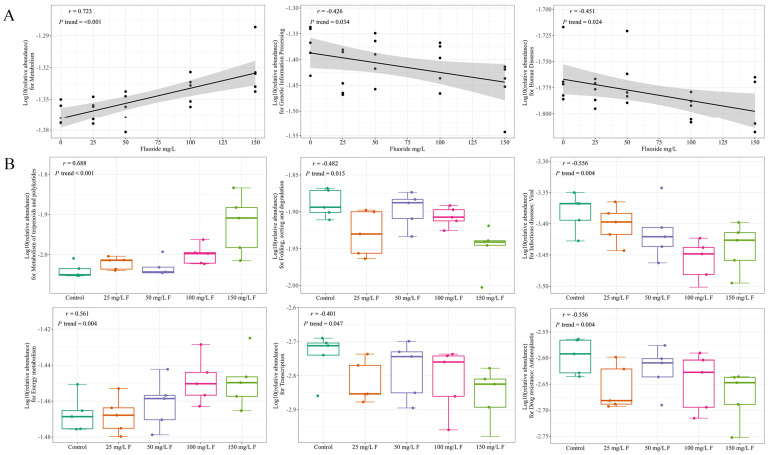
(**A**) The linear association between fluoride exposure and MetaCyc pathways of level 3. (**B**) Boxplots show the effect of fluoride exposure on the MetaCyc pathways of level 2 with Pearson correlation.

**Figure 6:**
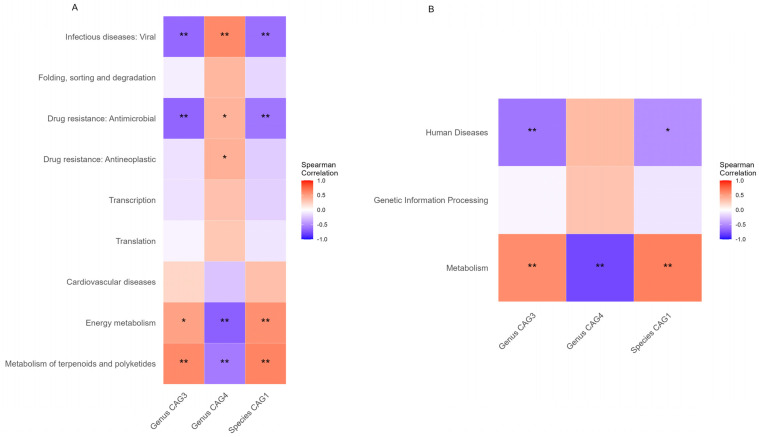
(**A**) Spearman correlations of the differentially abundant MetaCyc pathways of level 2 and CAGs; (**B**) Spearman correlations of the differentially abundant MetaCyc pathways of level 3 and CAGs. * indicates *p*-value < 0.05, ** indicates *p*-value < 0.01.

Figure 4 and Table S3 show that the biological functions of the gut microbiome were significantly associated with fluoride exposure. Metabolism pathways including energy metabolism and metabolism of terpenoids and polyketides were strongly positively correlated with fluoride exposure (Figure 5, Table 2 and Table 3) and strongly correlated with CAG3 in genus, CAG4 in genus, and CAG1 in species abundance (Figure 6). Furthermore, Genetic Information Processing pathways including Translation; Folding sorting, and degradation; and Transcription were negatively correlated with fluoride exposure (Figure 5, Table 2 and Table 3). Finally, Human Diseases pathways including drug resistance: antimicrobial, drug resistance: antineoplastic, and infectious diseases: viral were negatively correlated with fluoride exposure, while cardiovascular diseases was positively correlated (Figure 5, Table 2 and Table 3). Meanwhile, it also was negatively correlated with CAG3 in genus and CAG1 in species abundance (Figure 6).

The results of the predictions indicate that fluoride exposure exhibits a strong positive dose–response relationship (up-regulation) with functions like D-lyxose ketol-isomerase, alanine-synthesizing transaminase, Fis family transcriptional regulator, CoA-dependent NAD(P)H sulfur oxidoreductase, 3D-(3,5/4)-trihydroxycyclohexane-1,2-dione acylhydro-lase (decyclizing), and nickel transport system permease protein. Conversely, it displays a strong negative dose–response relationship (down-regulation) with functions like DNA polymerase III subunit gamma/tau, F-type H+-transporting ATPase subunit gamma, F-type H+-transporting ATPase subunit a, and F-type H+-transporting ATPase subunit c. Fluoride exposure has a pronounced positive dose–response relation (up-regulation) with metabolic pathways like metabolism of terpenoids and polyketides and energy metabolism, while it has a pronounced negative dose–response relation (down-regulation) with pathways like viral infectious diseases and folding, sorting, and degradation.

### 3.5. Associations between Functional Alterations and Gut Microbiome

The association analysis between functional alterations and the gut microbiome presented correlations ranging from −0.69 to 0.65. Significant positive correlations were found in Genus CAG3/Species CAG1 with MetaCyc pathways of level 2, such as metabolism of terpenoids and polyketides (Spearman’s correlation 0.60/0.62) and energy metabolism (0.49/0.57), and with MetaCyc pathways of level 3, such as metabolism (0.58/0.64). Meanwhile, a negative correlation was observed with MetaCyc pathways of level 2, such as drug resistance: antimicrobial (−0.67/−0.59) and infectious diseases: viral (−0.65/−0.62), and with MetaCyc pathways of level 3, such as human diseases (−0.59/−0.50) (Figure 6).

For the genus CAG4, significant positive correlations were found with MetaCyc pathways of level 2, such as infectious diseases: viral (Spearman’s correlation 0.60), drug resistance: antineoplastic (0.41), and drug resistance: antimicrobial (0.40), while a negative correlation was observed with MetaCyc pathways of level 2, such as energy metabolism (−0.69) and metabolism of terpenoids and polyketides (−0.68), and with MetaCyc pathways of level 3, such as metabolism (−0.79) (Figure 6).

In all, the three fluoride exposure-associated CAGs were significantly correlated with the metabolism of terpenoids and polyketides and thallium, energy metabolism, drug resistance: antimicrobial, and infectious diseases: viral (Figure 6A).

## 4. Discussion

In this study, we investigated the impact of varying concentrations of fluoride exposure on alterations in the gut microbial community and its functional changes. The research demonstrates significant dose–response changes in the composition of the gut microbiome in rats due to fluoride exposure. Moreover, these disturbed intestinal bacteria are closely associated with alterations in many gut microbiota-related functions, indicating that fluoride exposure not only interferes with the bacterial abundance but also significantly alters the functional characteristics of the gut microbiota, leading to a disruption in the host’s homeostasis post exposure.

The gut microbiota, including micro-organisms, their genomes, and the surrounding environment in the gut, has received unprecedented attention in the past decade [20]. Increasing evidence suggests that metabolic activities within the gut microbiota are intricately linked to human health and disease [21]. Several crucial functions executed by the gut microbiota are now widely recognized, encompassing polysaccharide digestion, biosynthesis of vitamins and nutrients, colonization resistance, and modulation of the immune system [21,22,23,24,25]. Moreover, the influence of the gut microbiota on the host’s metabolism and physiology extends beyond the intestine, impacting distant organs such as the liver, muscles, and brain [21,25]. Different gut bacteria have certain functions. For instance, *Bilophila* is conditionally pathogenic in healthy human hosts, which promotes intestinal inflammatory effects, intestinal barrier defects, systemic inflammation, bile acid dysmetabolism, and changes in the functional profile of the microbiome [26]. *Turicibacter* strains lead to changes in host bile acid profiles, and were positioned as modulators of host fat biology [27]. Roseburia has been proved to be a probiotic that can prevent intestinal inflammation and maintain energy homeostasis by producing metabolites [28]. The composition and function of the gut microbiota can be easily influenced by various intrinsic and extrinsic factors [29], and exposure to exogenous pollutants can lead to functional dysregulation of the gut microbiota [24,25,29]. More research is suggesting that the detrimental functional alterations in the gut microbiota due to exposure to these external pollutants might be associated with increased disease risk [21].

Numerous studies highlight that excessive fluoride exposure has adverse effects on human health, primarily resulting in dental and skeletal fluorosis [2] as well as unfavorable impacts on human reproductive development, the liver, kidney, endocrine system, nervous system, and genetics [2,3,4]. In recent years, there has been growing attention on soft tissue lesions associated with excessive fluoride exposure [30]. Studies have shown that fluoride metabolism is five times faster in rodents than in humans [31]. The fluoride doses used in the current study (25, 50, 100, and 150 mg/L NaF corresponding to 11.3, 22.6, 45.2, and 67.8 mg/L fluoride ion, respectively) are equal to environmental levels. Although the World Health Organization sets the guideline limit for fluoride in drinking water at 1.5 mg/L [32], the levels of fluoride in drinking water in endemic fluorosis areas are up to 16 mg/L [33]. Moreover, fluoride can be ingested from food and the air. Thus, the doses in this study were supposed to mimic the real human exposure in areas of endemic fluorosis. Research indicates that excessive fluoride exposure can impair the organism’s intestinal morphology and ultrastructure, leading to gastrointestinal diseases [12,34,35]. For instance, after exposure to 100 mg/L sodium fluoride for 90 days, C57BL/6J mice exhibited disrupted small intestine tissue structure and ultrastructural disorder, with a marked decrease in the ratio of villus height to crypt depth [36]. Exposure to 50 mg/L and 100 mg/L fluoride (calculated as fluoride ion) for 70 days resulted in severe structural damage to the rats’ colon and rectum, with a significant inhibition of epithelial cell proliferation [12,13]. However, the current research on the effects of fluoride exposure on the gut microbiota remains relatively limited [7,11,15]. Studies have found that environmental chemicals can directly impact gut bacteria by interrupting specific metabolic pathways or gene expressions, leading to differential selective pressures. This, in turn, reshapes the gut microbial community due to the unique set of metabolic pathways and genomes possessed by different bacterial species [37]. (Some environmental chemicals can directly affect the gut bacteria by interrupting specific metabolic pathways or gene expression, leading to distinct selection pressures, hence shaping the gut microbial community due to the uniqueness of the set of metabolic pathways and genome possessed by different bacterial species.) The potential mechanism behind the impact of fluoride exposure on the intestinal microbiota is likely related to the changes observed; however, the specific mechanisms remain unclear and warrant further research.

The current study indicates that exposure to fluoride induces significant dose-responsive alterations in the composition of the intestinal microbiome. Notably, different fluoride concentrations showed a positive correlation with CAG3 in different genera, with the genera *Bilophila*, *Holdemania*, *Pelagibacterium*, and *Ruminococcaceae* significantly positively correlated with fluoride concentrations. A plethora of studies demonstrate that there is a marked increase in the genus *Bilophila* among patients with diabetic nephropathy [38,39,40], IgA nephropathy [41], intrahepatic cholestasis of pregnancy [42], autism-related *Bilophila* abundance increase [43], and ovarian premature aging mice [44]. Additionally, an increase in both the *Bilophila* and *Ruminococcaceae* genera affects the reproductive performance of sows [45]. Chronic hepatitis B patients showed a correlation between an increase in the genus *Holdemania* and a heightened risk of chronic hepatitis B [46]. Elevated blood urea nitrogen levels in children with idiopathic nephrotic syndrome positively correlate with the *Ruminococcaceae* genus [47]. Moreover, patients with conditions such as diabetic nephropathy [38], liver–kidney transplantation [48], hepatitis C virus (HCV) infection [49], non-alcoholic fatty liver [50], liver fibrosis [51], polycystic ovary syndrome [52], thyroid cancer [53], Parkinson’s disease [54], autism spectrum disorder [55], multiple sclerosis [56], and spinal cord injury [57] all show a significant rise in the relative abundance of the *Ruminococcaceae* genus. After exposure to perfluorooctanesulfonic acid, there was a trend of increased abundance of *Ruminococcaceae*, but only the high-dose group was significantly higher than the control group [58]. This suggests that fluoride exposure-induced augmentation of the aforementioned intestinal microbes might further be associated with pathological changes in the kidneys, liver, and nervous system, reproductive development, and the thyroid.

In contrast, different fluoride concentrations were negatively correlated with CAG4 in different genera. The genera *Corynebacterium*, *Lachnospiracea incertae sedis*, *Roseburia*, and *Turicibacter* showed a significant negative correlation with fluoride concentrations. Recent studies revealed a significant decrease in the relative abundance of *Corynebacterium* in depression model rats compared to controls [59]. Patients with conditions such as polycystic ovary syndrome [60], IgA nephropathy [61], Parkinson’s disease [54], and non-alcoholic fatty liver [62] all display a marked reduction in the abundance of *Lachnospiracea incertae sedis*. Studies suggest that as the abundance of *Lachnospiracea incertae sedis* increases, levels of indolyl sulfate, formyl sulfate, and phenylacetylglutamine in circulation decrease, correlating with improved renal function [63]. Kidney diseases, including diabetic kidney disease (DKD) [64], chronic kidney disease (CKD) [65], and end-stage renal disease (ESRD) [66], are associated with a notable decrease in the Roseburia genus, potentially linked to its involvement in butyrate production, indole synthesis, and mucin degradation [67]. Autoimmune diseases show a marked reduction in *Roseburia* abundance in autoimmune disease mice [68], and patients with IgA nephropathy also exhibit a significant reduction in *Roseburia* compared to healthy individuals [62]. Neurological conditions such as schizophrenia [69], Parkinson’s disease [70], and spinal cord injury [71], as well as reproductive disorders like infertility, show a decrease in the number of *Roseburia* [72]. Compared to controls, benign prostatic hyperplasia rat groups displayed a marked reduction in *Turicibacter* [73], as did unilateral ureteral obstruction (UUO) rats [74]. This implies that fluoride exposure-induced diminishment of the aforementioned intestinal microbes might further correlate with pathologies in autoimmunity, the kidneys, liver, and nervous system, and reproductive development. The current study indicated that fluoride exposure causes a certain up-regulation of metabolic pathways like metabolism of terpenoids and polyketides and energy metabolism, while it causes a certain down-regulation of pathways like viral infectious diseases and folding, sorting, and degradation. Concurrently, strong correlations exist between these metabolic pathways and CAGs. In summation, these functional and metabolic pathway changes may represent potential mechanisms by which fluoride exerts toxicity by affecting the intestinal microbiota. In summation, our results suggest that the bacteria sensitive to fluoride in a fecal sample could be a biomarker of fluoride exposure, and these functional and metabolic pathway changes may be used to further explore the mechanism of bacteria in the pathogenesis of fluorosis.

The concept of intestinal microbiota toxicity has been increasingly recognized. Intestinal microbiota toxicity refers to the structural and functional changes in the gut microbial community caused by exposure to certain environmental chemicals, potentially leading to a series of adverse health consequences [75]. Environmental exposure is a significant risk factor for a range of human diseases that overlap with those associated with gut microbial composition [76,77]. Consequently, microbial community toxicity may represent the missing link between environmental exposure and microbial community-associated human diseases [75]. At present, this has not received adequate attention, and the dose–response relationship of gut microbiota toxicity has largely remained unexplored. In this study, we investigated the toxicity of fluoride on the gut microbiota and further explored the dose-response relationship between them.

## 5. Conclusions

We analyzed the effects of fluoride exposure at various concentrations on the gut microbial community and its functions in rats. Exposure to different concentrations of fluoride significantly altered the composition of the gut microbiota, showing a dose–response relationship with changes in the abundance levels of multiple bacterial genera. The functions and metabolic pathways of the gut microbiota experienced substantial alterations post fluoride exposure, also displaying a dose–response relationship. Moreover, correlation analyses identified that some gut bacterial CAGs (co-abundance groups) were highly associated with the altered metabolic pathways of the gut microbiota. In summary, fluoride exposure not only disrupted the gut microbiota composition in terms of abundance but also affected its functions and metabolic pathways. This study explored the pathogenesis of fluorosis and provided a clue for the biomarkers of fluoride exposure.

## Figures and Tables

**Table 1:** Relative abundance of the most abundant phyla in the rats’ gut microbiome composition (mean ± SD).

Phylum	Control	25 mg/L F	50 mg/L F	100 mg/L F	150 mg/L F	*F*	*p* Value
Firmicutes	81.12 ± 18.73	78.33 ± 9.95	75.97 ± 13.54	86.36 ± 6.66	80.84 ± 9.48	0.490	0.743
Bacteroidetes	17.71 ± 18.35	20.55 ± 9.75	23.09 ± 13.51	12.62 ± 6.76	17.94 ± 9.75	0.502	0.735
Proteobacteria	0.25 ± 0.11	0.35 ± 0.19	0.24 ± 0.12	0.35 ± 0.13	0.51 ± 0.12	2.995	0.043
Actinobacteria	0.31 ± 0.24	0.27 ± 0.12	0.24 ± 0.12	0.22 ± 0.09	0.29 ± 0.15	0.283	0.885

**Table 2:** Correlation coefficients between MetaCyc pathways of level 3 and fluoride exposure.

MetaCyc Pathways of Level 3	Correlation Coefficient	*p* Value
Metabolism	0.723	<0.001
Genetic Information Processing	−0.426	0.034
Human Diseases	−0.451	0.024

**Table 3:** Correlation coefficients between MetaCyc pathways of level 2 and fluoride exposure.

MetaCyc Pathways of Level 3	MetaCyc Pathways of Level 2	Correlation Coefficient	*p* Value
Genetic Information Processing	Translation	−0.399	0.048
Genetic Information Processing	Folding, sorting, and degradation	−0.482	0.015
Genetic Information Processing	Transcription	−0.401	0.047
Human Diseases	Drug resistance: Antimicrobial	−0.442	0.027
Human Diseases	Drug resistance: Antineoplastic	−0.411	0.041
Human Diseases	Infectious diseases: Viral	−0.556	0.004
Human Diseases	Cardiovascular diseases	0.399	0.048
Metabolism	Energy metabolism	0.561	0.004
Metabolism	Metabolism of terpenoids and polyketides	0.688	<0.001

## Data Availability

Data available on request due to restrictions eg privacy or ethica.

## Supplementary Materials

The following supporting information can be downloaded at https://www.mdpi.com/article/10.3390/metabo13111159/s1: Table S1: Correlation coefficient between gut microbiome in genera and fluoride exposure; Table S2: Correlation coefficient between gut microbiome in species and fluoride exposure; Table S3: Correlation coefficient between functional alterations and fluoride exposure.
