# Supplementary material for: The Dose–Response Effect of Fluoride Exposure on the Gut Microbiome and Its Functional Pathways in Rats
Source: Metabolites. 2023 Nov 17;13(11):1159. doi: 10.3390/metabo13111159 (PMC10672837; doi:10.3390/metabo13111159)
Supplement: Supplementary file 1 [file metabolites-13-01159-s001.zip › metabolites-2706178-supplementary.pdf]

metabolites

MDPI

Supplementary Information

The Dose–Response Effect of Fluoride Exposure on the Gut Microbiome and Its Functional Pathways in Rats

Zhe Mo [1,2,†], Jian Wang [1,†], Xinyue Meng [1], Ailin Li [1], Zhe Li [1], Wenjun Que [1], Tuo Wang [1], Korto Fatti Tarnue [1], Xu Ma [1], Ying Liu [1], Shirui Yan [1], Lei Wu [1], Rui Zhang [1], Junrui Pei [1,*] and Xiaofeng Wang [2,*]

[1] Key Lab of Etiology and Epidemiology, Education Bureau of Heilongjiang Province & National Health Commission (23618504), Institute for Fluorosis Disease Control, Center for Endemic Disease Control, Chinese Center for Disease Control and Prevention, Harbin Medical University, Harbin 150081, China

[2] Department of Environmental Health, Zhejiang Provincial Center for Disease Control and Prevention, Hangzhou 310051, China

* Correspondence: peijunrui@ems.hrbmu.edu.cn (J.P.); xfwang@cdc.zj.cn (X.W.)

† These authors contributed equally to this work.

Table. S1 Correlation coefficient between gut microbiome in genera and fluoride exposure.

Table. S2 Correlation coefficient between gut microbiome in species and fluoride exposure.

Table. S3 Correlation coefficient between functional alterations and fluoride exposure.

Table S1. Correlation coefficients between gut microbiome in genera and fluoride exposure.

Genus	CAGs	Correlation Coefficient	*p* Value
Allobaculum	3	0.150	0.474
Anaerofilum	3	0.217	0.298
Anaerofustis	3	0.038	0.857
Bilophila	3	0.501	0.011
Blautia	3	0.085	0.687
Clostridium XVIII	3	0.169	0.418
Erysipelotrichaceae incertae sedis	3	−0.007	0.973
Escherichia/Shigella	3	0.245	0.238
Eubacterium	3	0.179	0.392
Granulicatella	3	0.143	0.495
Holdemania	3	0.429	0.032
Pelagibacterium	3	0.677	<0.001
Rothia	3	0.376	0.064
Unclassified Bacillales	3	−0.248	0.232
Unclassified Bacteria	3	0.350	0.086
Unclassified Ruminococcaceae	3	0.497	0.011
Unclassified Streptococcaceae	3	0.182	0.383
Anaeroplasma	4	−0.238	0.252
Clostridium XlVb	4	−0.234	0.260
Clostridium sensu stricto	4	−0.320	0.119
Coprobacillus	4	−0.308	0.134
Corynebacterium	4	−0.434	0.030
Lachnospiracea incertae sedis	4	−0.447	0.025
Lactonifactor	4	−0.173	0.408
Paenalcaligenes	4	−0.284	0.169
Roseburia	4	−0.479	0.015
Turicibacter	4	−0.490	0.013
Unclassified Desulfovibrionaceae	4	−0.262	0.205

Table S2. Correlation coefficients between gut microbiome in species and fluoride exposure.

Species	CAGs	Correlation Coefficient	*p* Value
Acetatifactor muris	1	0.362	0.075
Bacteroidales bacterium ph8	1	0.534	0.006
Bacteroides fragilis (T)	1	0.507	0.010
Bacteroides merdae	1	0.222	0.287
Bacteroides uniformis	1	0.448	0.025
Candidatus Soleaferrea massiliensis	1	0.652	<0.001
Eubacterium coprostanoligenes (T)	1	0.145	0.490
Gram-negative bacterium cL10-2b-4	1	0.197	0.346
Parabacteroides distasonis	1	0.617	0.001
Parabacteroides goldsteinii	1	0.478	0.016
Unclassified Alistipes	1	0.144	0.491
Unclassified Allobaculum	1	0.150	0.474
Unclassified Alphaproteobacteria	1	0.456	0.022
Unclassified Bacteroidetes	1	0.091	0.665
Unclassified Bdellovibrionales	1	0.586	0.002
Unclassified Butyricimonas	1	0.144	0.494
Unclassified Clostridiales	1	0.165	0.429
Unclassified Erysipelotrichaceae incertae sedis	1	0.267	0.198
Unclassified Escherichia/Shigella	1	0.245	0.238
Unclassified Eubacterium	1	0.220	0.291
Unclassified Odoribacter	1	0.504	0.010
Unclassified Parasutterella	1	−0.032	0.879
Unclassified Pelagibacterium	1	0.677	<0.001
Unclassified Prevotellaceae	1	−0.110	0.599
Unclassified Ruminococcaceae	1	0.566	0.003
Unclassified Ruminococcus	1	0.193	0.357
Bacterium YE57	1	0.147	0.484
Uncultured Alkalibacter sp.	1	−0.079	0.709
Uncultured Bacillales bacterium	1	−0.154	0.462
Uncultured Bacteroidetes bacterium	1	0.327	0.110
Uncultured Desulfovibrionaceae bacterium	1	0.426	0.034
Uncultured Elusimicrobium sp.	1	−0.095	0.651
Uncultured Erysipelotrichales bacterium	1	0.432	0.031
Uncultured Kopriimonas sp.	1	0.504	0.010
Uncultured bacterium adhufec108	1	0.610	0.001
Uncultured eubacterium WCHB1-54	1	0.173	0.410
Unidentified bacterium	1	0.105	0.618

Table S3. Correlation coefficients between functional alterations and fluoride exposure.

Function	Correlation Coefficient	*p* Value
amino-acid N-acetyltransferase [EC:2.3.1.1]	0.765	<0.001
pyruvate kinase [EC:2.7.1.40]	−0.768	<0.001
allophanate hydrolase [EC:3.5.1.54]	0.769	<0.001
adenylosuccinate synthase [EC:6.3.4.4]	−0.762	<0.001
F-type H+-transporting ATPase subunit a	−0.771	<0.001
F-type H+-transporting ATPase subunit b	−0.766	<0.001
F-type H+-transporting ATPase subunit c	−0.771	<0.001
F-type H+-transporting ATPase subunit epsilon	−0.760	<0.001
F-type H+-transporting ATPase subunit gamma	−0.783	<0.001
cobalamin biosynthetic protein CobC	0.765	<0.001
DNA polymerase III subunit gamma/tau [EC:2.7.7.7]	−0.788	<0.001
3D-(3,5/4)-trihydroxycyclohexane-1,2-dione acylhydrolase (decyclizing) [EC:3.7.1.22]	0.775	<0.001
Fis family transcriptional regulator, factor for inversion stimulation protein	0.778	<0.001
tRNA(Ile)-lysidine synthase [EC:6.3.4.19]	−0.756	<0.001
ribosome biogenesis GTPase / thiamine phosphate phosphatase [EC:3.6.1.-3.1.3.100]	−0.760	<0.001
uncharacterized protein	0.752	<0.001
adenine-specific DNA-methyltransferase [EC:2.1.1.72]	−0.758	<0.001
MerR family transcriptional regulator, mercuric resistance operon regulatory protein	0.752	<0.001
D-lyxose ketol-isomerase [EC:5.3.1.15]	0.818	<0.001
D-xylose transport system permease protein	0.757	<0.001
hydrogenase-4 component B [EC:1.-.-.-]	0.751	<0.001
two-component system, cell cycle sensor histidine kinase and response regulator CckA [EC:2.7.13.3]	0.767	<0.001
alanine-synthesizing transaminase [EC:2.6.1.-]	0.790	<0.001
nickel transport system permease protein	0.772	<0.001
succinate-semialdehyde dehydrogenase [EC:1.2.1.76]	0.753	<0.001
CoA-dependent NAD(P)H sulfur oxidoreductase [EC:1.8.1.18]	0.776	<0.001
D-psicose/D-tagatose/L-ribulose 3-epimerase [EC:5.1.3.30 5.1.3.31]	0.768	<0.001
cob(I)alamin adenosyltransferase [EC:2.5.1.17]	0.754	<0.001
nicotine oxidoreductase [EC:1.5.3.-]	0.757	<0.001
diguanylate cyclase [EC:2.7.7.65]	0.757	<0.001
